# Erratum: Case Report: Focal Myoclonus with a Striatal Lesion as a Presentation of Subacute Sclerosing Panencephalitis

**DOI:** 10.4269/ajtmh.22-0046err

**Published:** 2022-12-14

In “Case Report: Focal Myoclonus with a Striatal Lesion as a Presentation of Subacute Sclerosing Panencephalitis” by Kalita and others (https://doi.org/10.4269/ajtmh.22-0046), one of the author’s names is misspelled. The correct spelling of the second author’s name is Sarvesh K. Chaudhary.

